# Robotic First Rib Resection in Thoracic Outlet Syndrome: A Systematic Review of Current Literature

**DOI:** 10.3390/jcm12206689

**Published:** 2023-10-23

**Authors:** Maikerly Reyes, Sneha Alaparthi, Johannes B. Roedl, Marisa C. Moreta, Nathaniel R. Evans, Tyler Grenda, Olugbenga T. Okusanya

**Affiliations:** 1 Sidney Kimmel Medical College, Thomas Jefferson University, Philadelphia, PA 19107, USA; mxr513@students.jefferson.edu; 2 Department of Thoracic Surgery, Thomas Jefferson University Hospital, Philadelphia, PA 19107, USA; sneha.alaparthi@jefferson.edu (S.A.); nathaniel.evans@jefferson.edu (N.R.E.); tyler.grenda@jefferson.edu (T.G.); 3 Department of Radiology, Thomas Jefferson University Hospital, Philadelphia, PA 19107, USA; Johannes.roedl@jefferson.edu; 4 Department of Rehabilitation Medicine, Thomas Jefferson University Hospital, Philadelphia, PA 19107, USA; Marisa.moreta@jefferson.edu

**Keywords:** Robotic First Rib Resection, thoracic outlet syndrome, neurogenic thoracic outlet syndrome, arterial thoracic outlet syndrome, venous thoracic outlet syndrome

## Abstract

Thoracic outlet syndrome (TOS) involves the compression of neurovascular structures in the thoracic outlet. TOS subtypes, including neurogenic (nTOS), venous (vTOS), and arterial (aTOS) are characterized by distinct clinical presentations and diagnostic considerations. This review explores the incidence, diagnostic challenges, and management of TOS with a focus on the innovative approach of Robotic First Rib Resection (R-FRR). Traditional management of TOS includes conservative measures and surgical interventions, with various open surgical approaches carrying risks of complications. R-FRR, a minimally invasive technique, offers advantages such as improved exposure, reduced injury risk to neurovascular structures, and shorter hospital stays. A comprehensive literature review was conducted to assess the outcomes of R-FRR for TOS. Data from 12 selected studies involving 397 patients with nTOS, vTOS, and aTOS were reviewed. The results indicate that R-FRR is associated with favorable intraoperative outcomes including minimal blood loss and low conversion rates to traditional approaches. Postoperatively, patients experienced decreased pain, improved function, and low complication rates. These findings support R-FRR as a safe and effective option for medically refractory TOS.

## 1. Introduction

Thoracic outlet syndrome (TOS) comprises a host of conditions that involve neurovascular compression of the brachial plexus, subclavian artery, or vein in the region between the lower neck and the axilla. The overall incidence of this condition ranges from 3 to 80 per 1000 [1]. Neurogenic TOS is the most common subtype and comprises about 90% to 95% of TOS cases [2]. The remaining cases are of vascular etiology, with venous being more common than arterial [1]. Initial management consists of physical therapy, nonsteroidal anti-inflammatory drugs (NSAIDs), weight reduction, and thrombolytics [3]. However, in cases of nonsurgical management failure, including progressive neurologic dysfunction or vascular insufficiency, thoracic outlet decompression surgery is indicated. The mainstay of treatment is first rib resection and scalenectomy via a variety of approaches including transthoracic, transaxillary, supraclavicular, and infraclavicular [4]. One literature review cites a rate of 5% nerve injury in the transaxillary approach in addition to the supraclavicular approach, compared with a 3% rate in video-assisted thoracic surgery (VATS) [5]. Open surgical approaches are associated with a risk of neurovascular complications and incomplete resection of the more medial part of the first rib. Furthermore, first rib resection and scalenectomy are the greatest sources of malpractice claims against cardiothoracic surgeons due to nerve injury [6]. 

More recently, minimally invasive approaches via video-assisted and robotic-assisted thoracoscopic surgery have become more common for first rib resection due to better exposure of the first rib as well as improved avoidance of injury to critical structures such as the brachial plexus, subclavian artery, and phrenic nerve [7]. Robotic First Rib Resection (R-FRR) is a relatively new and innovative surgical procedure for the treatment of TOS, first described in 2012 for the treatment of Paget-Schroetter disease by Gharagozloo and his group in the George Washington University Medical Center [4]. Robotic First Rib Resection allows for the complete resection of the offending part of the first rib. The procedure is groundbreaking due to instrument dexterity in confined spaces and a high-definition 3D view of the operative field [8]. In this study, we review the literature to investigate the indications for a R-FRR referral as well as if R-FRR should be one of the new primary modalities for approaching patients who have not found symptomatic relief with nonoperative management. 

### 1.1. Diagnosis and Types of TOS

Thoracic outlet syndrome describes a group of conditions involving compression of the brachial plexus and the subclavian vasculature by the first rib, scalene muscles, and the clavicle [9,10]. TOS is classically categorized into three types according to which structure is predominantly affected: neurogenic, arterial, and venous (Table 1). Neurogenic makes up the majority of cases (90–95%) followed by venous (3–5%), and then arterial (1%) [2,11]. While TOS shares some symptoms, subtypes may be differentiated by certain physical exam findings and diagnostic imaging. However, there are no pathognomonic clinical features for subtypes of TOS (Table 1). 

### 1.2. Neurogenic TOS (nTOS)

Brachial plexus compression defines neurogenic TOS, and symptoms include but are not limited to neuropathic pain, sensory loss, paresthesia of the fingers in the ulnar nerve distribution, weakness of the upper extremity, neck pain, and occipital headaches [12,13]. Patients may also show a diminished pulse in the wrist and Raynaud’s phenomenon, which may falsely lead to incorrect diagnosis of arterial TOS [13,14]. Symptoms can often be exacerbated or reproduced when patients engage in activities involving exertion. A common etiology includes scarred scalene muscles due to neck trauma such as whiplash [13]. Severe presentation of nTOS may demonstrate a Gilliatt–Sumner hand, which is defined by thenar, hypothenar, and intrinsic hand muscle atrophy [14]. 

Diagnosis of nTOS is clinical, as there is no highly sensitive or specific single test. Various physical exam maneuvers may be used to reproduce symptoms in nTOS, including palpation over the scalene muscles to assess for tenderness, rotation of the neck and tilting the head from ear to shoulder, abduction of the arms to ninety degrees, and the upper limb tension test [13]. While the upper limb tension test is not pathognomonic for nTOS, it has been found to elicit positive symptoms in up to 98% of patients with TOS. Other provocative maneuvers include the Adson and the elevated arm stress test (EAST). Unfortunately, there is no gold standard diagnostic test for the condition and no established diagnostic criteria. As a result, many patients experience a delay in correct diagnosis and may undergo unnecessary procedures. 

Furthermore, diagnosis of nTOS is challenging, due to a multitude of musculoskeletal culprits that can mimic the condition [15]. Certain shared features have been identified in patients with nTOS, such as particular anatomy, a history of trauma, and occupations with frequent use of the upper limbs. Accessory ribs, short costoclavicular distance, and a protuberant C7 process are examples of particular anatomy which places patients at higher risk of nTOS [14]. Diagnostic imaging such as Duplex US may help narrow down the diagnosis and exclude vTOS and aTOS. Chest and neck radiographs should be considered in all forms of TOS to evaluate for bony abnormalities [1]. MRI may help rule out cancer or infectious and inflammatory etiologies and differential diagnoses such as disk herniation or spinal stenosis. CT and EMG may be helpful as well. While these imaging tests may play an important role in those patients with an equivocal diagnosis, they are often normal or nonspecific for nTOS. There is increasing evidence for the utility of neuromuscular blockade of the scalene muscles as a diagnostic, therapeutic, and prognostic tool for the effectiveness of surgical decompression. 

### 1.3. Venous TOS (vTOS)

Venous TOS occurs due to subclavian vein compression and is characterized by upper limb edema with pain, venous distension, cyanosis, and risk of thrombosis of the arm due to compression of the subclavian vein in the costoclavicular space [16]. This is most commonly secondary to anatomic narrowing of the costoclavicular triangle due to hypertrophy of the anterior scalene and subclavius muscle or as a result of trauma to the anterior chest among other causes [17]. Arm swelling and cyanosis are strong indicators of venous TOS [13]. Additional symptoms may include pain and paresthesia, although paresthesia is due to edema rather than actual nerve compression as observed in nTOS. Additionally, vTOS carries significant morbidity such as risk of thrombosis and pulmonary emboli. 

Imaging is vital in the diagnosis of vTOS to confirm or support the diagnosis as well as to detect the cause of subclavian vein compression and any complications [17]. Like in nTOS, a chest radiograph is useful to detect the presence of accessory ribs or first rib anomalies and to rule out cancerous lesions. Still, chest radiographs have low sensitivity, and their role is for screening only [17]. Duplex US (DUS) is typically the initial test of choice and has a sensitivity and specificity of 78–100% and 82–100%, respectively. DUS allows for the assessment of venous flow alterations throughout different arm movements. However, DUS does carry limitations, such as the inability to detect rare etiologies of vTOS like lung malignancy as well as limited visibility due to osseous structures causing shadowing. 

Advanced imaging such as CT and MRI venography can accurately identify the area of venous narrowing and the extent of thrombosis with and without provocative upper limb maneuvers [17]. Catheter-directed venography is the gold standard for vTOS diagnosis, allowing for precise detection of narrowing of the venous lumen, thrombi, and extent of obstruction [17]. 

### 1.4. Arterial TOS (aTOS)

Arterial TOS arises due to emboli or aneurysms in the subclavian artery from repetitive trauma, and patients exhibit symptoms of ischemia, paresthesia, claudication, and pallor in the digits and hand, and it more commonly occurs in patients with bony abnormalities [13]. Notably, symptoms are less likely to be present in the neck or shoulder. Furthermore, patients may demonstrate an absent radial pulse. It may be differentiated from nTOS by the absence of scalene muscle tenderness and lack of positive symptoms on physical exam maneuvers like neck rotation and head tilt as well as the upper limb tension test. aTOS presentation may be variable, with acute thrombosis, chronic stenosis, and total occlusion, among others [18]. 

Diagnostic imaging includes neck radiographs, CT, MRI/MRA, and Duplex US. aTOS has bony and non-bony causes which can be detected by imaging. Bony causes include an accessory rib, bony abnormalities due to trauma, and bone malignancy [18]. Nonbony etiologies of aTOS include scalene hypertrophy and arterial disease, among others. Catheter arteriography is the gold standard to assess for aTOS as it clearly shows subclavian artery compression as well as other findings such as subclavian artery aneurysm, thrombus, clot, and dissection. 

### 1.5. Current Treatment and Management

**Figure 1:**
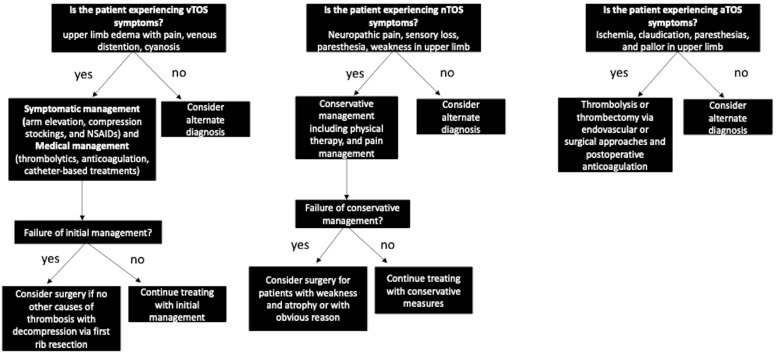
Decision Tree Based on Type of TOS.

Due to the various types of TOS, there is wide variability in terms of outcomes concerning both conservative and surgical management, and as a result, a paucity of evidence with regard to best management practices [15]. Additionally, the treatment of TOS varies slightly based on the underlying structure being compressed. A decision tree based on the type of TOS is outlined in Figure 1. 

nTOS is initially managed conservatively with pain management and physical therapy. Management algorithms have suggested that patients with satisfactory improvement in nTOS symptoms should remain on physical therapy with follow-up, while surgery should be reserved for patients with weakness and atrophy who fail to improve from physical therapy or those with an otherwise obvious reason for surgery (such as a bony abnormality) [14,15,19]. Other treatment algorithms have suggested instead that only patients with improvement after physical therapy should undergo surgery [19]. Dengler et al., 2022 state that surgery should rarely be indicated for patients that have diffuse or solely cervicoscapular symptoms and no anatomic anomaly [14]. 

In vTOS, factors include the severity of venous compression and symptoms as well as the absence or presence of thrombosis guide management. Initial therapeutic goals include relief of upper limb pain and swelling, and prevention of pulmonary embolism [17]. Symptomatic management includes arm elevation, compression stockings, and NSAIDs. Medical management of vTOS includes thrombolytics and anticoagulation. More invasive management involves catheter-based treatments to dissolve the clot and has been associated with decreased rates of post-thrombotic syndrome and relief of acute symptoms. However, the success of catheter-based interventions is highly dependent on early diagnosis and intervention within the first two weeks. In vTOS, surgery is typically indicated for those without other causes of axillosubclavian venous thrombosis (past central venous catheter, hypercoagulable state) and includes surgical decompression via first rib resection, debulking of the subclavius and anterior scalene muscles, and venolysis [20]. 

Evidence for conservative management of aTOS is limited due to disease rarity. Studies have shown that half of patients may benefit from at least 6 months of therapy [21]. Moreover, unlike other subtypes, aTOS typically requires thrombolysis or thrombectomy via endovascular or surgical approaches with anticoagulation postoperatively. When considering surgery for aTOS, it is important to take into consideration whether vascular reconstruction is necessary when planning an approach. 

### 1.6. Surgery for Treatment of TOS

Surgery for the treatment of TOS remains controversial. Despite most patients reporting relief of symptoms, patients still experience persistent functional impairments as measured by the Quick Disability of the Arm, Shoulder, and Hand Survey (DASH) [22]. Still, both perioperative complications and mortality are rare and the annual volume of TOS operations in the U.S. has shown an increasing trend from 2203 in 2010 to 3092 cases in 2015 [15]. 

Surgical decompression of the thoracic outlet can involve a variety of extrathoracic approaches such as the transthoracic, supraclavicular, infraclavicular, transaxillary, or thoracoscopic approach [1]. All surgical approaches carry risks of injury and impairment, such as neurovascular compromise as well as incomplete first rib resection and incomplete decompression of the subclavian vein. The transaxillary approach with first or accessory rib resection may be complicated by injury to the brachial plexus, sympathetic chain, subclavian vein, and thoracic duct. Patients may also develop pneumothorax. Complications arise due to challenges of exposure and access in extrathoracic surgical approaches. The minimally invasive VATS approach is an alternate technique that allows for clear visualization of the top surface of the rib and operative field, although with limited maneuverability of surgical instruments. The VATS technique offers magnified views and does not require retraction of components in the neurovascular bundle, thereby reducing the risk of brachial plexus injury. Studies of the VATS approach in TOS patients have found favorable outcomes with no mortalities, major complications, recurrence at follow-up, and up to 90% of patients with complete resolution of symptoms [23]. Additionally, TOS patients surgically treated with VATS have shown similar recovery rates as traditional transaxillary approaches [24]. The complication rate of VATS for TOS has been reported to be between 3–25%. Complications reported are due to wound infections, pneumothorax, and brachial plexus injuries, among others [5]. 

VATS for the treatment of TOS has been further innovated by the use of robotic surgical systems. While the use of robotic surgical systems is relatively novel in first rib resection for TOS, they improve upon extrathoracic and thoracoscopic approaches via 3D high definition and improved maneuvering of instruments in the surgical area of interest. Gharagozloo et al., 2012 were the first to describe Robotic First Rib Resection for the treatment of vTOS in a series of four men and one woman using four incisions for ports and access with a reported mean length of stay of three days [4]. 

The debate surrounding the less expensive VATS versus R-FRR remains difficult to resolve, as demonstrated by lung cancer surgery [25]. Still, R-FRR can improve upon VATS approaches via improved exposure, accessibility, and better exposure of the first rib [25]. The use of purely endoscopic treatments poses a limit to VATS approaches, while the robotic system offers advantages such as endo-wrist movement and improved visualization. As a result, robotic systems improve ergonomics, allowing thoracic surgeons to work in narrow spaces while maintaining high-quality visibility of critical neurovascular structures. To our knowledge, there are no studies showing a clear advantage of VATS over R-FRR for the treatment of TOS. Therefore, randomized prospective trials are needed to evaluate for differences in clinical outcomes between VATS and R-FRR, if any exist. 

## 2. Methods

### 2.1. Search Strategy

We used Pubmed, the Cochrane database of systematic reviews, and Embase to conduct a literature search of all published studies during 2012–2023 investigating Robotic First Rib Resection for TOS. The keywords used in the search were “thoracic outlet syndrome” AND “robotic first rib” OR “thoracic outlet syndrome” AND “RATS (robotic-assisted thoracic surgery)” OR “thoracic outlet syndrome” AND “VATS”. 

### 2.2. Study Selection

Studies were independently reviewed by two reviewers, SA and MR. Articles were eligible for inclusion if the following criteria were met: studies describing outcomes of patients diagnosed with TOS after R-FRR and human studies in English with available full text. Exclusion criteria included reviews, meta-analyses, case reports, and studies with duplicate data. Studies were assessed with regards to symptom resolution, mean operative time, complications, and length of stay. 

## 3. Results

**Figure 2:**
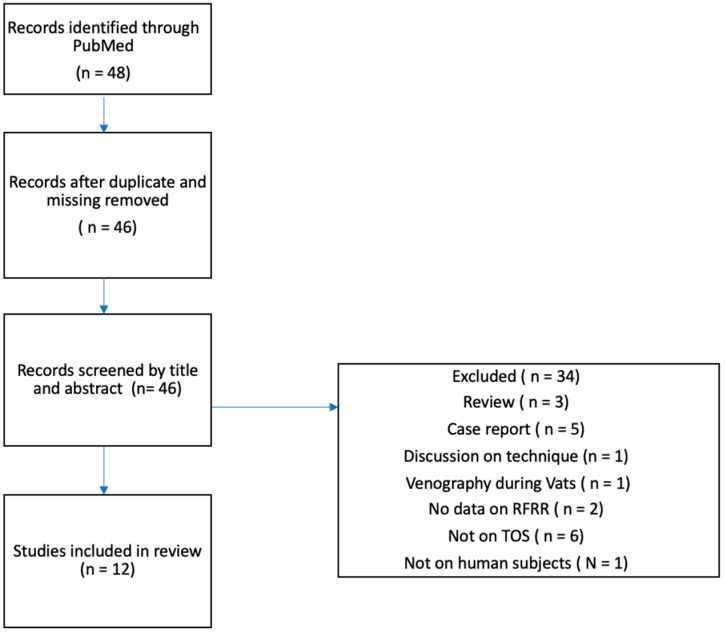
Flow diagram of study selection.

Forty-eight articles matched our initial search criteria (Figure 2). After review of the articles by our two reviewers, only 12 articles fit our inclusion criteria for this review. 

### 3.1. Prevalence of Robotic-First Rib Resection for Different TOS Subtypes

A total of 397 patients underwent R-FRR and there were 522 R-FRR completed (Table 2). The majority of patients that underwent R-FRR had vTOS (283), followed by nTOS (188) and aTOS (22). This overall represents 36% cases of nTOS, 4.2% of aTOS, 54.2% of vTOS, and 4.5% cases of nonspecific TOS. 

### 3.2. Demographics Data: Age and Gender

Of the studies that included breakdown by gender in their cohorts, 55.8% were found to be female and 44.2% male (Table 2). Age breakdown was included in 10 studies, of which the average age was 33.86. The highest median age in one study was 47 ± 11 years, while the lowest was 24 ± 8.5 in two studies.

### 3.3. Symptom Laterality and Postoperative Pain Scores

Out of the reviewed studies, eight included a breakdown of laterality of TOS symptoms. Among these studies, there were 167 cases of right-handed dominant TOS, 78 cases of left-handed dominant, and 13 bilateral. Our findings revealed that most R-FRR procedures were performed on patients’ dominant sides. Two studies indicated that right-handed patients underwent surgery on their right side with frequencies ranging from 72.2–75.4%. DASH scores were only collected in two studies, where preoperative scores ranged from a median of 60–64.2. One study included a preoperative VAS Score. 

### 3.4. Intraoperative Outcomes after R-FRR

To evaluate intraoperative outcomes, we assessed variations in the duration of surgery, complications, and estimated blood loss (EBL). Operative time ranged from a median of 87.6 ± 10.8 to 195 ± 24.6 min (Table 2). This was reported in 11 studies. Among these, the average operative time was 133 min. Only three intraoperative complications were reported and all consisted of conversion to a transaxillary approach. Two studies reported quantitative estimated blood loss. One study reported a mean EBL of 20 mL, while the other reported 79.5 mL. Intraoperative variables and postoperative outcomes are detailed in Table 3.

### 3.5. Operative Outcomes after TOS

Eleven studies included data on median postoperative length of stay (LOS). Of these, the mean LOS was found to be 2.57 days. This ranged from 1.8 ± 1.9 days to 4 days. Postoperative complications were seen in five studies. Nine postoperative complications were reported. These complications included phrenic nerve injury, brachial plexus palsy, sensory injury, pneumonia, pleural effusion (primary and recurrent), hemothorax, pneumothorax requiring chest tube, and postoperative hematoma. One study reported a preoperative visual analog scale (VAS) score of 6.0 ± 2.5 that decreased to 2.8 ± 2.1 and 1.4 ± 1.5 at the first and second postoperative visit, respectively. Postoperative VAS score was included in five studies, of which the range was from 0.6–4.7%. Mean preoperative DASH scores were reported in two studies with nTOS patients as 60.3±2.1 and 64.2 ± 17.1. DASH scores were reported postoperatively in three studies. These were also broken down into scores at serial postoperative visits. 

## 4. Discussion

Thoracic outlet syndrome remains a challenging disease from diagnosis to treatment. Traditional surgical therapy risks several complications including major vascular injury, chyle leak, brachial plexus injury, phrenic nerve injury, and chronic pain. Therefore, innovative approaches to avoid injury during FRR are critical. 

### Evidence for R-FRR for the Treatment of TOS

Prior to minimally invasive approaches, first rib resection has been achieved via open surgical approaches such as the transthoracic, transaxillary, supraclavicular, and infraclavicular approaches. A study comparing the supraclavicular approach with R-FRR found that while SC-FRR and R-FRR did not differ with regards to length of procedure and hospital stay, R-FRR had lower rates of brachial plexus palsy, total less frequent complications, lower postoperative pain as measured by the VAS score, and decreased use of opioids [7]. R-FRR has been found to have rates of brachial plexus injury ranging from 0–3.4% [7]. Compared to a SC-FRR, R-FRR has also been found to be associated with lower VAS scores and greater improvements in VAS scores after surgery [26]. 

R-FRR is safe and associated with a short LOS. In fact, among our selected studies there were only three intraoperative complications reported, which consisted of conversion to the transaxillary approach. No other intraoperative complications were reported. Postoperatively, only nine complications were reported among the selected studies out of 503 patients. This may be because R-FRR allows for improved exposure and complete resection of the first rib compared to traditional open surgical approaches, without inadvertent injury to critical neurovascular structures such as the brachial plexus, subclavian vein, subclavian artery, and phrenic nerve [7]. Blood loss during R-FRR is also minimal and was reported as 20 mL and 79.5 mL [7,27]. 

Moreover, R-FRR has been reported to have positive postoperative outcomes. For patients with nTOS, the immediate mean postoperative DASH score was reported to be 5.5 ± 2.3 and 3.5 ± 1.1 at the 6-month postoperative visit, indicating a notable reduction in the level of disability after surgery. In another study, preoperative mean VAS and DASH scores decreased from 6.0 and 64.2 to 2.8 and 35.0, respectively [26]. In patients with vTOS, patency of the subclavian vein after 15.5 months of follow-up was reported to be 91% with great functional outcomes as measured by DASH scores at one and three years postoperatively at 7.1 and 6.0, respectively [27]. Collectively, these studies show significant improvement in pain symptoms and function. Finally, in another study evaluating R-FRR in vTOS and aTOS, 18 out of 24 patients reported complete resolution of symptoms while 6 out of 24 reported partial relief at follow-up [25]. Rates of symptom relief in the literature for TOS after R-FRR ranged from 91–100% in comparison to 90% symptom relief via the open transaxillary approach, and 90% symptom relief after the VATS approach [2,8,22,25,28]. Although symptom relief rates are similar, future prospective multicenter studies are needed to truly evaluate differences in rates of symptom relief after TOS surgery.

Another consideration is the ease of teaching the procedure to the next generation of surgeons. The robotic approach has the benefit of excellent visualization of the entire field and the ability to pass on limited progressive portions of the operation to the trainee. Open approaches, especially the axillary approach, can be challenging to teach due to the limited views. As robotic skills become more prominent in the training of all surgeons, there may be a comfort in approaching this disease from that vantage.

Improved accuracy during R-FRR as well as short LOS, decreased rates of intraoperative and postoperative morbidity, as well as significant gains in functional outcome point to R-FRR as a formidable option for surgical management of all types of TOS. More studies are needed comparing open surgical approaches and VATS with R-FRR to determine if R-FRR should become the new standard of care for refractory TOS cases. Additionally, prospective studies assessing VAS and DASH scores pre- and postoperatively would help determine the long-term impact on functional outcomes in TOS after R-FRR. 

## 5. Conclusions

Minimally invasive R-FRR is a safe and effective method in the treatment of medically refractory TOS. R-FRR offers technical advantages over traditional open approaches as well as decreased rates of injury to the brachial plexus and complications. R-FRR also offers greater improvement in functional outcomes. More studies comparing R-FRR to standard surgical approaches are needed to evaluate if R-FRR should be the new standard of care for surgical management of TOS. 

## Figures and Tables

**Table 1:** TOS Overview and Classification.

TOS Classification	Predominant Affected Structure	Key Symptoms	Diagnostic Methods
**Neurogenic TOS (nTOS)**	**Brachial plexus**	- Neuropathic pain—Sensory loss—Paresthesia of ulnar nerve distribution—Weakness of upper extremity—Neck pain—Occipital headaches—Diminished pulse—Raynaud’s phenomenon—Gilliatt-Sumner hand (severe cases)	- Clinical diagnosis—Physical exam maneuvers (scalene tenderness, neck rotation, head tilt, upper limb tension test, Adson test, EAST)—Imaging (Duplex US, chest and neck radiographs, MRI, CT, EMG)—Neuromuscular blockade of scalene muscles
**Venous TOS (vTOS)**	**Subclavian vein**	- Upper limb edema—Pain—Venous distension—Cyanosis—Risk of thrombosis	- Imaging (Duplex US, chest radiograph, CT, MRI venography, catheter-directed venography)
**Arterial TOS (aTOS)**	**Subclavian artery**	- Ischemia—Paresthesia—Claudication—Pallor in digits and hand—Absent radial pulse	- Imaging (neck radiographs, CT, MRI/MRA, Duplex US, catheter arteriography)

**Table 2:** Perioperative Characteristics of Patients Undergoing Robotic First Rib Resection for TOS.

PMID:	Number of Patients	R-FRR Cases	nTOS	aTOS	vTOS	Non-Specific TOS	Females	Males	Age (Years)	R Handed	Left Sided	Bilateral	Preoperative VAS Score	Preoperative DASH Score
34795965	162	162	79	0	83	0	84	78	24 ± 8.5 (vTOS) 34 ± 9.5 (nTOS)	N/A	N/A	N/A	N/A	60.3 ± 2.1 (nTOS)
34592270	34	38	4	3	20	11	21	13	32.5	N/A	9	4	N/A	N/A
33046231	66	72	50	0	22	0	47	19	36	59	28	6	N/A	N/A
33832359	15	15	0	0	15	0	7	8	32.9 (mean)	N/A	6	0	N/A	N/A
35350142	11	11	1	2	8	0	N/A	N/A	28	N/A	3	0	N/A	N/A
29696329	8	8	4	0	3	0	4	4	N/A	N/A	2	0	N/A	N/A
36327061	42	47	5	7	22	13	24	18	47 (mean)	N/A	16	0	N/A	N/A
32446920	17	17	8	0	9	0	9	8	45 ± 11 (mean)	N/A	N/A	N/A	N/A	N/A
30085044	83	83	0	0	83	0	34	49	24 ± 8.5 Y (mean)	N/A	N/A	N/A	N/A	N/A
34501401	23	24	0	10	13	0	16	7	N/A	N/A	6	0	N/A	N/A
22576034	5	5	0	0	5	0	1	4	34.6 ± 10	N/A	N/A	N/A	N/A	N/A
34419432	37	40	37	0	0	0	32	5	36	34	8	3	6.0 ± 2.5	64.2 ± 17.1

**Table 3:** Intraoperative and Postoperative Outcomes in Patients with TOS who Underwent R-FRR.

PMID:	Blood Loss	Operative Time (minutes)	Complications	LOS	Postoperative Complications	VAS Score	DASH Score
34795965	N/A	87.6 ± 10.8 (nTOS), 127.6 ± 20.8 min (vTOS)	none	3 days (nTOS), 4 days (vTOS)	none (nTOS)	N/A	5 ± 2.3 (immediate post-op nTOS), 3.5 ± 1.1 (nTOS 6 months), N/A vTOS
34592270	N/A	133 ± 44.7 min	none	2 ± 2.1 days	none	N/A	N/A
33046231	20 mL	140		47.8 h	5 (phrenic n injury, brachial plexus palsy, sensory, any)	4.7	N/A
33832359	79.5 mL	147.9 min (mean)	3 (conversion to transaxillary)	3.5 days (mean)	pneumonia, pleural effusion, hemothorax		short term: general 7.1, work 5.8, sport/recreation 13.1; long-term: general 6, work 5.4, sport/recreation 12.5
35350142	N/A	180 min (median)	none	2 days (median)	1 (pneumothorax requiring chest tube)	0.6 (mean)	N/A
29696329	not significant	108 min (median)	none	2 days	none	N/A	N/A
36327061	N/A	122 ± 40 min	none	3 ± 1 days	2 (pneumothorax requiring chest tube, recurrent pleural effusion)	2 (SD = 1)	N/A
32446920	N/A	113.2 ± 55.3 min (mean)	N/A	1.8 ± 1.9 days (mean)		N/A	N/A
30085044	N/A	127 min ± 20.8 min	none	4 days (median)	none	N/A	N/A
34501401	N/A	117	N/A	2 days	1 (postop hematoma)	3	
22576034	N/A	195 ± 24.6 min	none	3 days (median)	NONE	N/A	N/A
34419432	N/A	N/A	N/A	N/A	N/A	1st postop = 2.8 ± 2.1, 2nd post-op = 1.4 ± 1.5	1st post op = 35.0 ± 21.3, 2nd post-op = 30.2 ± 19.8

## Data Availability

No new data were created or analyzed in this study. Data sharing is not applicable to this article.
